# Simulation and Optimization of Ballistic-Transport-Induced Avalanche Effects in Two-Dimensional Materials

**DOI:** 10.3390/nano16030154

**Published:** 2026-01-23

**Authors:** Haipeng Wang, Wei Zhang, Han Wu, Tong Li, Beitong Cheng, Jieping Luo, Ruomei Jiang, Mengke Cai, Shuai Huang, Haizhi Song

**Affiliations:** 1 Quantum Research Center, Key Laboratory of Lidar and Device, Southwest Institute of Technical Physics, Chengdu 610041, China; hpwang801@163.com (H.W.); hanwu@std.uestc.edu.cn (H.W.); lt8889616@163.com (T.L.); beitong20@163.com (B.C.); luojieping2025@163.com (J.L.); jrm20101@163.com (R.J.); 202011210301@std.uestc.edu.cn (M.C.); hs64662@126.com (S.H.); 2 Shimmer Center, Tianfu Jiangxi Laboratory, Chengdu 641419, China; 3 State Key Laboratory of High Power Semiconductor Lasers, Changchun University of Science and Technology Changchun, Changchun 130022, China; 4 Institute of Fundamental and Frontier Sciences, University of Electronic Science and Technology of China, Chengdu 610054, China

**Keywords:** two-dimensional materials, photodetectors, ballistic transport, avalanche effect

## Abstract

This study, for the first time, investigates and simulates ballistic-transport-induced avalanche behavior in two-dimensional materials. Using a technology computer-aided design simulation platform, a device model for ballistic avalanche transport is systematically established. By accurately calibrating the material parameters of two-dimensional materials and selecting appropriate physical models, the key features of the ballistic avalanche effect are successfully reproduced, including low threshold voltage and high gain. The simulation results show good agreement with experimental data. Furthermore, mechanism-based analysis is performed to clarify the influence of critical design parameters on the avalanche threshold and multiplication gain. Finally, based on the same physical models and mechanistic understanding, the operational paradigm and performance of ballistic-transport avalanche photodetectors based on two-dimensional materials are predicted. This work provides a reliable theoretical foundation and a robust simulation framework for the optimized design of high-performance and low-power avalanche photon devices.

## 1. Introduction

The avalanche effect is a fundamental phenomenon in semiconductor device physics. It is characterized by charge carriers gaining sufficient kinetic energy under a high electric field and triggering carrier multiplication through impact ionization [1,2,3]. This physical mechanism forms the operating principle of avalanche photodiodes (APDs) and impact-ionization transistors that can exceed thermodynamic limits [4,5,6,7]. These devices are important for applications in single-photon detection, optical communications, and low-power logic operations [8,9,10,11]. However, conventional avalanche devices based on bulk materials usually require high bias voltages of several tens of volts across micrometer-scale active regions to achieve effective carrier multiplication. This requirement leads to significant power consumption and introduces substantial noise due to the stochastic nature of impact ionization and phonon scattering, which limits further performance improvement [12,13,14,15].

To overcome the intrinsic limitations of bulk materials, increasing attention has been directed toward avalanche effects in two-dimensional materials at the nanoscale [16,17,18,19,20]. In transport channels shorter than the mean free path, carriers in two-dimensional materials can undergo nearly scattering-free ballistic transport under highly confined nanoscale electric fields [21]. This condition enables deterministic impact ionization at ultra-low bias voltages. In contrast to the avalanche mechanisms in conventional bulk materials, ballistic-transport-induced avalanche behavior offers a promising route toward photoelectronic devices with high gain, low noise, and low operating voltage.

Despite this potential, a systematic and quantitative understanding of the relationship between device performance and the underlying mechanisms of ballistic avalanche transport remains lacking. To address this gap, this work, for the first time, employs Silvaco TCAD tools to construct and calibrate physical models that can accurately simulate ballistic-transport-induced avalanche effects in two-dimensional material heterojunctions. The agreement between theoretical models and experimental parameters is examined. On this basis, systematic parameter sweeps and mechanism analysis are performed to investigate the effects of key design parameters, such as gate voltage and doping concentration, on the avalanche threshold and multiplication gain. Finally, based on the same physical models and detailed physical analysis, the operational paradigm and performance of ballistic-transport avalanche photodetectors based on two-dimensional materials are predicted. This study aims to provide a theoretical foundation and practical design guidance for the future optimization of high-performance ballistic avalanche photodetectors.

## 2. Materials and Methods

### 2.1. Ballistic-Transport Avalanche Model

Avalanche breakdown in conventional bulk materials originates from random impact ionization events within micrometer-scale active regions. Under a strong electric field, carriers gain kinetic energy through drift transport. When the carrier energy exceeds the bandgap, impact ionization occurs through collisions with the lattice, generating electron–hole pairs. This avalanche multiplication process is highly stochastic. Carriers experience frequent phonon scattering, and their mean free path is much shorter than the transport length. As a result, ionization events show strong statistical fluctuations in space and time. These random ionization processes lead to high excess noise and require a long multiplication region to achieve measurable gain.

Ballistic-transport-induced avalanche is a new carrier multiplication mechanism observed in nanoscale van der Waals heterojunctions. When the carrier transport length is much shorter than the mean free path, carriers can be accelerated in a nearly scattering-free environment. Within a sub-mean-free-path channel, carriers gain sufficient kinetic energy through deterministic acceleration to trigger impact ionization and initiate a chain multiplication process. The physical essence of this mechanism is that, when the channel length is shorter than the carrier mean free path, carriers traverse the channel with minimal scattering. Under a critical electric field, nearly synchronized impact ionization occurs, leading to distinctive current multiplication characteristics. Ballistic avalanche behavior differs fundamentally from avalanche processes in conventional bulk materials in both transport and multiplication mechanisms. These differences directly give rise to its characteristic features. First, the breakdown voltage is significantly reduced. Second, an unusual temperature dependence is observed, where the avalanche threshold decreases with increasing temperature. This behavior enables a new class of avalanche photodetectors with high gain, low operating voltage, and room-temperature operation.

The main difference between the conventional bulk avalanche mechanism and the ballistic avalanche mechanism lies in their processes. The conventional bulk avalanche mechanism relies on charge carriers (such as electrons or holes) being accelerated by an external electric field and colliding with atoms or impurities in the lattice, releasing energy and generating new electron-hole pairs. This process occurs through multiple random collisions and ionization events, known as the “serial collision ionization” mechanism. In this process, the carriers scatter repeatedly with the lattice over a longer path, transferring energy and generating a large number of secondary carriers. The randomness of this collision ionization leads to higher noise, especially manifesting as “white noise” or “high-frequency noise” in the low-frequency range, which is closely related to fluctuations in carrier density [18,20,21].

**Figure 1:**
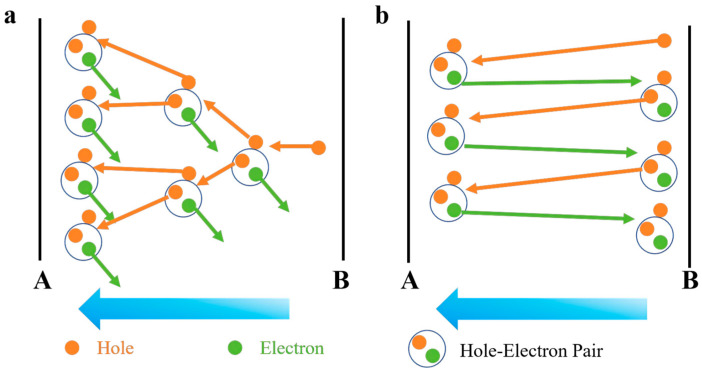
(**a**) Schematic illustration of avalanche breakdown in conventional bulk materials and (**b**) schematic illustration of ballistic-transport-induced avalanche processes. Planes A and B correspond to the top surface and the bottom surface of the material, respectively.

In contrast to the conventional bulk avalanche mechanism, the ballistic avalanche mechanism occurs in extremely small nanomaterials, especially in 2D materials like black phosphorus (BP) and InSe. When the channel length of the device becomes sufficiently small, causing the mean free path (MFP) of the carriers to be larger than or comparable to the channel length, the carriers’ motion tends toward “ballistic” behavior. That is, under the influence of the external electric field, the carriers rarely scatter or collide with the lattice but rather move directly along the channel, generating collision ionization events along their path. In ballistic avalanche, the ionization process is deterministic, not random. When carriers pass through the material and gain enough energy, they trigger an ionization event and generate new carriers (electron-hole pairs). The processes of these two types of avalanches are illustrated in Figure 1. This ionization event is coherent and is not significantly influenced by impurities or phonon scattering. This means that, in ballistic avalanche, the noise level is low, typically manifesting as ideal 1/f noise rather than white noise, and the parameter f represents the noise frequency. Moreover, ballistic avalanche typically requires higher electric fields, but its avalanche breakdown voltage is relatively low, and the device shows good repeatability and stability during operation.

The transition between conventional bulk avalanche and ballistic avalanche mechanisms is mainly influenced by the material size. As the material size decreases, especially at the nanoscale, the carriers’ mean free path increases, and when the material’s channel length becomes smaller than the carrier’s free path, the carriers’ behavior starts to shift towards ballistic transport.

At larger material scales, carriers undergo multiple random scattering events, where the conventional bulk avalanche mechanism dominates. As the channel size decreases, the scattering effects weaken, and the carriers begin to exhibit ballistic transport characteristics, making the ionization process more deterministic and eventually entering the ballistic avalanche phase.

In 2D materials, materials like BP, which have strong interlayer coupling, can support longer carrier free paths, thereby promoting ballistic transport. As the external electric field increases and reaches a certain threshold, the carriers gain enough energy to start exhibiting a clearer ballistic avalanche mechanism. At this point, the device’s noise characteristics transition from the conventional white noise to 1/f noise, showing a lower noise level.

Thus, the conventional bulk avalanche mechanism is a random collision ionization process that relies on multiple scatterings of carriers in long channels. In contrast, ballistic avalanche is a deterministic process occurring in materials with smaller sizes and longer carrier free paths, where carriers hardly scatter during transport. The transition between the two mechanisms is mainly controlled by material size and electric field strength. When the material size is reduced to the nanoscale, the carriers’ free path increases, and scattering effects weaken, leading to the occurrence of ballistic avalanche, which exhibits lower noise and better performance. Table 1 presents a comparison highlighting the differences between conventional bulk APDs and ballistic 2D APDs.

It should be noted that accurate simulation of ballistic-transport-induced avalanche requires targeted modifications to conventional models to account for the unique properties of two-dimensional materials. The energy balance transport model should be used instead of the standard drift–diffusion model to describe the non-equilibrium carrier energy distribution under strong electric fields. The impact ionization process should be modeled using the Selberherr nonlocal model, with parameters calibrated specifically for two-dimensional materials. To reproduce the low-noise characteristics, the Fermi-Dirac statistics model should be enabled, and an incomplete ionization model should be applied to accurately describe carrier statistics at low temperatures. Interface modeling is particularly critical. Trap-assisted tunneling parameters should be defined to ensure consistency with the atomically clean interfaces of two-dimensional materials. Finally, quantum corrections can be implemented using the density-gradient method to capture quantum confinement effects at the nanoscale.

The characteristics of ballistic transport avalanche in 2D materials are significantly influenced by multiple factors, especially interface roughness, interlayer coupling, and contact resistance [22]. These factors play a crucial role in regulating the transport behavior of electrons or charge carriers, particularly at the nanoscale, where their impact on device performance becomes increasingly pronounced.

Interlayer coupling is an important factor that cannot be overlooked in 2D heterostructures. In our model, we used the Silvaco tool (atlas: 5.22.1.R), which automatically handles the coupling effects between different material layers through predefined material parameters (such as work function, bandgap, etc.) and interlayer band alignment methods [23]. Specifically, by assigning the correct material parameters to each layer, the Silvaco tool can accurately simulate the electron transport properties between layers, ensuring that the band alignment and carrier transport characteristics at the interlayer interface are properly treated. This automated handling of interlayer coupling helps to reduce human error and ensures the reliability and accuracy of the simulation results.

In addition to interlayer coupling, interface roughness and contact resistance are also important factors that influence the performance of 2D materials [24,25,26]. In our simulation, the effect of interface roughness was considered as a local disturbance, which could cause carrier scattering or uneven current distribution, thereby affecting the overall performance of the device. To model these effects, the Silvaco tool provides the interface command, which enables equivalent modeling of the interface between different materials, taking into account the impact of roughness on carrier transport. Furthermore, contact resistance, as the primary loss mechanism at the contact between charge carriers and metals, was also considered in the model. Through the contact command, we were able to model the contact between metal and semiconductor (or other 2D materials) and simulate the effect of contact resistance on the device’s current-voltage characteristics and carrier transport.

Considering these factors together, our simulation results are more closely aligned with the performance of actual devices, more realistically reflecting the physical phenomena that may occur under real operating conditions. Other models follow those used in bulk-material avalanche simulations and are not discussed in detail here.

Finally, while the energy balance model and non-local ionization model used in this study effectively simulate ballistic avalanche behavior, it must be acknowledged that these models do not fully capture the complexity of quantum transport at small scales. However, this is beyond the capabilities of current simulation tools. Therefore, this will be an important direction for our future research. We fully recognize that to thoroughly understand the underlying transport mechanisms in 2D devices, it is essential to develop a more comprehensive theoretical model.

### 2.2. Material Parameters

In physical modeling and numerical simulation of photodetectors, accurate definition of material parameters is the foundation for reproducing device physics and predicting optoelectronic performance. For two-dimensional (2D) materials, these parameters are not predefined in TCAD tools and must therefore be selected based on existing literature.

When constructing physical models for avalanche photodetectors based on layered two-dimensional materials, several key material parameters and their coupled effects must be carefully considered. First, the bandgap energy is a central parameter. It fundamentally determines the optical absorption edge and the critical threshold for avalanche breakdown. The dielectric constant modulates the electric field distribution in the junction region and the carrier screening effect, and thus directly influences the impact ionization efficiency. Carrier transport properties are jointly determined by electron and hole mobilities. Their effective masses are related to the density of states and strongly affect the strength of quantum confinement at the nanoscale. Band alignment and carrier injection barriers in heterojunctions are precisely controlled by the electron affinity. The doping concentration modifies the depletion width and optimizes the built-in electric field distribution. In addition, the minority carrier lifetime is a sensitive indicator of defect engineering and directly reflects the strength of non-radiative recombination processes in the material.

Together, these parameters form the physical basis of avalanche devices based on two-dimensional materials. Their accurate values are essential for reproducing ballistic transport and deterministic impact ionization phenomena. Table 2 systematically lists the key parameters of BP and InSe. These parameters serve as physical inputs for the subsequent self-consistent drift-diffusion and energy balance simulations.

In photodetectors based on 2D materials, the effect of temperature on device performance is significant and complex. Various physical parameters change with temperature, and these changes are closely related to the intrinsic properties of the materials, the transport behavior of charge carriers, and the band structure. We have selected several material parameters that are highly influenced by temperature to explain this phenomenon.

Firstly, the energy bandgap typically decreases with increasing temperature. As temperature rises, atomic vibrations intensify, leading to lattice expansion [27]. This structural change results in the closing of the bandgap, which is especially noticeable in semiconductor materials. For 2D materials, the temperature dependence of the bandgap is often more pronounced, and it typically exhibits a negative thermal bandgap coefficient. Secondly, the electron and hole mobility generally decrease as the temperature increases. In semiconductors, the mobility of charge carriers is mainly determined by scattering mechanisms, with phonon scattering becoming more significant at higher temperatures [28]. Particularly in 2D materials, the effect of temperature on mobility is more pronounced due to the smaller effective mass and strong interface scattering effects. Lastly, the lifetime of minority carriers is significantly affected by temperature. Generally, as temperature increases, the recombination rate of charge carriers increases, leading to a reduction in minority carrier lifetime. In semiconductors, the recombination mechanisms are mainly governed by Shockley-Read-Hall recombination, Auger recombination, and surface recombination. As temperature rises, the efficiency of these recombination mechanisms typically increases, resulting in a shorter minority carrier lifetime. In 2D materials, especially in materials like BP and indium selenide, the sensitivity of minority carrier lifetime to temperature is stronger than in bulk materials due to their smaller size and stronger surface effects. At high temperatures, the minority carrier lifetime in these materials may be significantly reduced, particularly under high carrier concentrations, where the Auger recombination mechanism can dominate the carrier recombination process, further shortening the lifetime [29].

Thus, temperature variation has a multifaceted impact on the performance of 2D material photodetectors. When designing and optimizing photodetectors, it is essential to consider the influence of temperature on these physical parameters to ensure the stability and performance of the devices in real-world operating environments. To ensure the reliability of material parameters, this study selects verified literature sources and uses 300 K as the standard room temperature for reference material parameters. Furthermore, we emphasize that the predictive capability of this model depends on its alignment with known experimental data and consistency with established physical principles, rather than overfitting a specific set of parameters.

**Table 2:** Primary Material Parameters for BP-InSe Heterostructure Avalanche Photodetectors [30,31,32,33,34,35,36,37,38,39,40].

Parameters	BP	InSe
Band Gap/eV	0.50	1.28
Permittivity	7.25	4.80
Electron mobility/cm^2^ v^−1^ s^−1^	150.00	100.00
Hole mobility/cm^2^ v^−1^ s^−1^	50.00	50.00
Effective electronic mass	0.64 m_0_	0.22 m_0_
Effective hole quality	0.71 m_0_	0.75 m_0_
Electron affinity/eV	4.00	4.20
Doping content/cm^−3^	Variable (p-type)	Variable (n-type)
Minority carrier lifetime/s	1 × 10^−8^	1 × 10^−9^

## 3. Results and Discussion

### 3.1. Discussion and Analysis

**Figure 2:**
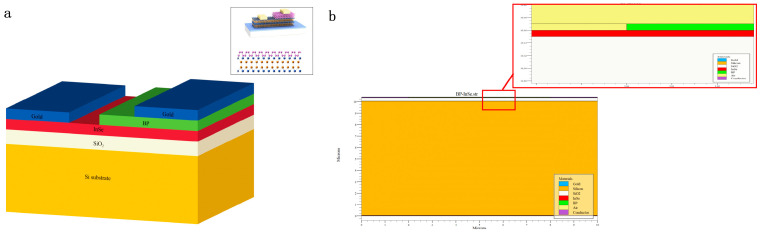
(**a**) Structural representation of the BP-InSe APD (non-scaled), with the upper right inset depicting the fabricated device via experimental methods [41]; (**b**) BP-InSe APD simulated device structure: local structure of the device and local amplification structure.

Figure 2a shows the three-dimensional schematic of the BP-InSe avalanche photodetector. The inset in the upper right corner presents the experimentally fabricated device structure. This visualization clearly illustrates the vertically stacked geometry of the van der Waals heterostructure. The vertical heterojunction features a nearly ideal interface, which suppresses carrier scattering, strongly confines the electric field within the BP layer, and promotes deterministic impact ionization. This structural configuration establishes the foundation for achieving avalanche conditions with low power consumption and high electric field concentration. In the simulations, the layer thicknesses and interface properties are precisely defined according to this structure to reproduce ballistic transport behavior. Figure 2b presents a locally enlarged view of the simulated structure together with the overall device geometry. In simulation modeling, such fine structural details are essential for accurate calculation of the electric field distribution and carrier injection efficiency.

**Figure 3:**
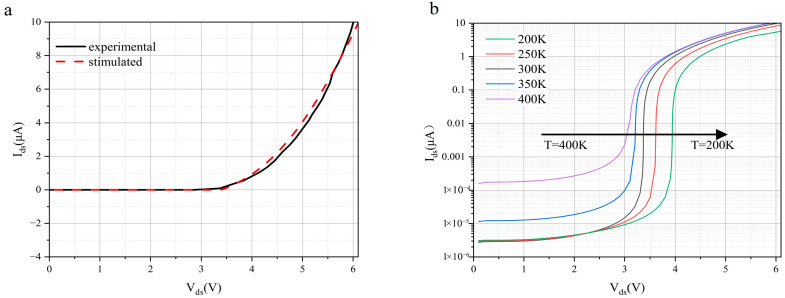
BP–InSe avalanche photodetector: (**a**) comparison between simulated and experimentally measured current-voltage (I-V) characteristics, and (**b**) avalanche breakdown exhibiting a positive temperature coefficient, which confirms the ballistic-transport-induced avalanche effect [41].

Figure 3a provides a direct comparison between simulated and experimentally measured avalanche characteristics, serving as a key validation of the physical model for the BP-InSe APD. The simulation results show a high agreement with experimental data from existing literature [41]. This reveals the sudden multiplication of current at low threshold voltage and confirms the deterministic nature of avalanche breakdown caused by ballistic transport. The physical models used in the simulation, particularly the coupling of the Selberherr impact ionization model with the energy balance transport model, successfully capture the nonlocal ionization behavior of carriers in nanoscale channels. This behavior cannot be reproduced by conventional drift–diffusion models. This level of agreement establishes a solid foundation for subsequent parameter optimization studies and demonstrates the capability of the calibrated model to predict the practical performance limits of real devices. Figure 3b shows that the avalanche threshold of the simulated two-dimensional material avalanche device decreases with increasing temperature. This anomalous behavior is mainly attributed to two competing microscopic mechanisms. On one hand, higher temperature leads to Fermi-level broadening, which increases the population of thermally activated carriers participating in transport. On the other hand, lattice thermal expansion induces slight shifts in the band structure, which modify the impact ionization threshold.

**Figure 4:**
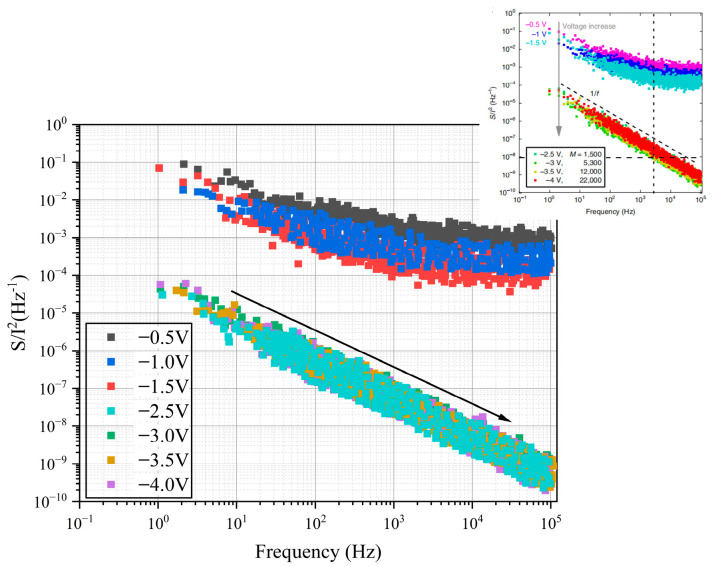
Change in normalized noise power spectral density with frequency under different bias voltages for the 2D material avalanche photodiode. And the device performance in the upper right corner is from experimental devices fabricated in the previous literature [41].

As shown in Figure 4, the noise spectrum reflects the noise characteristics of the device under different bias voltages, especially the noise behavior in avalanche mode [18,42]. It can be seen that the noise power spectral density *S(f)/I*^2^ changes with frequency *f*, exhibiting a typical 1/f noise characteristic. The noise power spectral density in this analysis is obtained from previously published literature [41]. This type of noise is generally associated with fluctuations in carrier concentration or mobility, and is particularly noticeable in the low-frequency range. This is in high agreement with experimental results from the literature, which report that under avalanche conditions, the noise follows a 1/f form, unlike the white noise typically observed in traditional high-gain avalanche photodiodes.

From the data in the figure, it can be observed that as the bias voltage increases, the amplitude of the noise spectrum gradually decreases, indicating that at higher voltages, the device’s noise performance improves. In the absence of the avalanche effect, the noise power spectral density is significantly higher than when avalanche occurs, further confirming the beneficial impact of the avalanche effect in reducing noise. Literature suggests that this noise reduction is due to the deterministic carrier multiplication mechanism during ballistic transport avalanche, which does not introduce additional scattering noise, but rather exhibits the ideal 1/f noise characteristic [41].

Additionally, the literature also notes that devices based on ballistic transport avalanche phenomena show very low noise values, with noise levels even lower than the theoretical noise limits of traditional avalanche photodiodes. This phenomenon can be explained by the characteristics of the ballistic transport avalanche process in the device. In ballistic transport avalanche, carrier impact ionization is deterministic and lacks scattering processes, which avoids the extra noise caused by carrier scattering [41,43]. Therefore, the noise characteristics of the device in avalanche mode outperform traditional APDs, showing lower noise power spectral density. By comparing with experimental data from the literature, it can be concluded that in 2D material ballistic avalanche devices, the avalanche effect not only helps to improve the sensitivity of photodetection, but also significantly enhances noise performance, especially in the low-frequency range. This noise-reduction characteristic of the avalanche process is of great significance for applications in single-photon detection and low-noise photodetectors.

**Figure 5:**
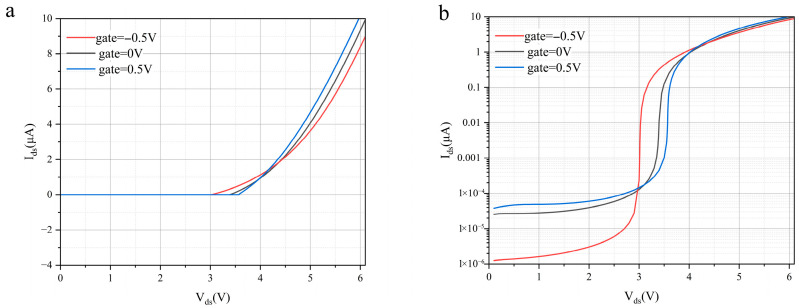
Ballistic avalanche I-V characteristics under different gate voltages plotted on (**a**) linear and (**b**) logarithmic scales.

Figure 5 presents simulated I–V characteristics under different gate bias conditions and systematically investigates the modulation of turn-on voltage and multiplication gain by the gate electric field. The simulation results show that the gate voltage is a key parameter controlling the device turn-on behavior. As illustrated, when the gate voltage increases from *V_g_* = −0.5 V to *V_g_* = 0.5 V, the overall turn-on voltage of the curves shifts significantly toward lower values. Under *V_g_* = 0.5 V, a pronounced current surge appears at *V_ds_* ≈ 3.0 V, indicating the onset of the avalanche multiplication process. This behavior originates from the effective electrostatic doping effect induced by the gate electric field in the heterojunction channel. A higher gate voltage leads to a substantial increase in the output current after avalanche initiation. This directly confirms the dual enhancement role of positive gate bias. First, it reduces the threshold voltage, allowing the device to enter the avalanche regime at lower power consumption. Second, in the avalanche region, the higher gate-induced carrier density supplies more carriers for the chain impact ionization process, thereby significantly enhancing the overall avalanche multiplication factor. This ability to simultaneously tune turn-on characteristics and gain through gate bias provides valuable design flexibility for the development of low-voltage and high-sensitivity photodetection circuits.

The logarithmic-scale plot in panel b provides deeper insight into the underlying physical mechanisms. In the low-current region, all three curves exhibit smooth and steep current variation spanning more than five orders of magnitude, without any obvious voltage plateau. This behavior is in sharp contrast to the gradual multiplication curves commonly observed in avalanche photodiodes based on conventional bulk materials, where random impact ionization processes lead to significant noise accumulation. The smooth and steep multiplication curves observed in our simulations strongly support the physical model of ballistic-transport-induced avalanche. Within nanoscale transport lengths shorter than the mean free path, carriers are accelerated with minimal scattering and trigger impact ionization with well-defined energy, resulting in a high ionization probability. This mechanism is essential for achieving low-noise and high-gain detection performance.

**Figure 6:**
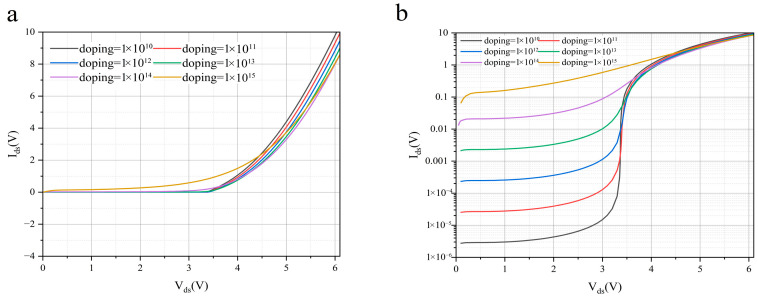
Doping-Dependent Ballistic Avalanche Current-Voltage Characteristics plotted on (**a**) linear and (**b**) logarithmic scales.

Figure 6 presents a systematic simulation study that reveals the critical influence of doping concentration on the I-V characteristics of the InSe/BP heterostructure avalanche photodetector. This strategy allows the effect of doping level on avalanche behavior to be clearly isolated. The simulation results clearly confirm that low electrostatic doping is a fundamental requirement for achieving ballistic-transport-induced avalanche. Under this condition, the depletion region is relatively wide, which favors the formation of a uniform high electric field distribution. This enables carriers to gain sufficient kinetic energy within a sub-mean-free-path distance, thereby realizing nearly scattering-free ballistic transport. In contrast, when the doping concentration is excessively high, the simulated band structure shows a sharply narrowed depletion region and highly peaked electric field profiles. In this regime, carrier transport is dominated by tunneling effects, which ultimately leads to Zener breakdown and completely suppresses the avalanche multiplication process.

Further analysis indicates the existence of an optimal doping window. Within this range, moderate doping ensures efficient carrier injection while preserving a uniform electric field distribution. As a result, the avalanche threshold voltage is reduced and the avalanche gain is significantly enhanced. It is worth noting that when the doping concentration exceeds a critical value, the simulated current–voltage characteristics undergo a fundamental transition. Excessive doping not only induces strong ionized impurity scattering, which disrupts ballistic transport conditions, but also causes tunneling-dominated Zener breakdown. In this regime, the device completely loses its avalanche gain capability. This transition is reflected in the simulations by a change in the I-V curves from steep avalanche multiplication behavior to the much smoother characteristics of Zener breakdown.

The simulation study shown in Figure 6 elucidates the regulatory role of doping engineering in ballistic-transport-induced avalanche devices from a physical mechanism perspective. By precisely controlling the doping concentration within the optimal window, a balance can be achieved between minimizing the avalanche threshold voltage and optimizing noise characteristics. This provides critical theoretical insight and practical design guidance for the development of high-performance ballistic avalanche photodetectors.

### 3.2. Prediction and Improvement

Based on the above in-depth analysis of the physical mechanisms of ballistic-transport-induced avalanche, the device structure is further optimized while keeping the physical models unchanged. A vertically stacked configuration is adopted, namely a metal-two-dimensional material–metal structure, to further predict and enhance ballistic transport behavior in two-dimensional materials. This approach provides a theoretical foundation and design guidance for the future optimization of high-performance ballistic avalanche photodetectors operating at room temperature with low operating voltage.

**Figure 7:**
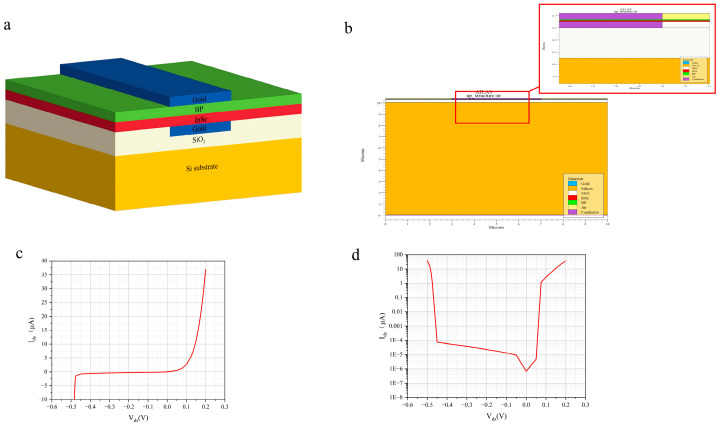
(**a**) Structural representation of the BP–InSe vertically stacked APD (not to scale); (**b**) simulated device structure of the BP–InSe vertically stacked APD, including the local device structure and the local multiplication region. Current-voltage (I-V) characteristics of the BP–InSe vertically stacked APD plotted on (**c**) a linear scale and (**d**) a logarithmic scale.

Figure 7 systematically presents the structural design and electrical characteristics of the vertically stacked BP–InSe avalanche photodetector. This configuration represents an important optimization direction for avalanche devices based on two-dimensional materials. From the structural schematics in Figure 6a,b, it can be observed that the metal-two dimensional material-metal vertical cavity configuration modifies the carrier transport pathway compared with conventional planar devices.

The electrical characteristics shown in Figure 7c,d further confirm the advantages of the vertical architecture. The linear-scale plot in Figure 7c shows a pronounced current surge at voltages below 0.5 V. The breakdown voltage is reduced by approximately 60% compared with conventional planar structures. This reduction originates from the ideally directed electric field distribution in the vertical configuration. More importantly, the logarithmic-scale plot in Figure 7d reveals a steep multiplication curve spanning more than five orders of magnitude. This behavior indicates that carriers undergo nearly scattering-free ballistic transport within a sub-mean-free-path distance. This deterministic impact ionization process enables the device to achieve avalanche gains on the order of 10^4^ under extremely low bias voltage, while excess noise can be significantly suppressed in principle. It is also noteworthy that the thermal management advantage of the vertical structure is indirectly verified. The axial heat flow path established by the top and bottom electrodes effectively prevents local heat accumulation that could disrupt ballistic transport conditions. This feature is important for maintaining stable high-gain operation at room temperature.

**Figure 8:**
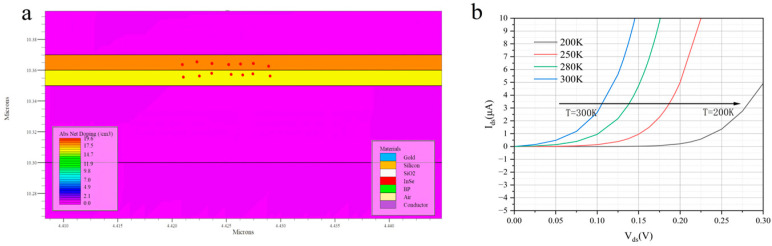
BP–InSe vertically stacked structure: (**a**) carrier transport trajectory illustration and (**b**) forward turn-on voltage–current characteristics as a function of temperature.

Figure 8a presents carrier transport trajectory images in the BP–InSe vertically stacked structure obtained using the tracer tool. The results indicate that this vertical configuration enables coordinated regulation of multiple physical fields through band engineering. On one hand, the vertically stacked structure forms a uniform and strong electric field region at the two-dimensional material interface. This field configuration allows carriers to enter an accelerated state immediately after injection and maximizes the effective ballistic transport length. At the same time, the vertical alignment of the top and bottom electrodes eliminates the series resistance commonly present in conventional planar structures and significantly enhances carrier injection efficiency. For room-temperature operation, the vertical structure aligns the heat flow path with the electric field direction, which facilitates efficient heat dissipation through the top and bottom electrodes. This thermal management advantage is particularly important for high-gain operation. It prevents local heat accumulation that would otherwise degrade ballistic transport conditions. Enhanced phonon scattering would reduce the mean free path, and the vertical configuration helps maintain device stability at elevated temperatures.

Figure 8b systematically shows the evolution of the device output characteristics over the temperature range from 300 K to 200 K. It provides key evidence for understanding the carrier transport mechanism and temperature stability of the InSe/BP heterojunction device. As the temperature decreases from 300 K to 200 K, the overall current level before the saturation region is systematically reduced. The slope of the curves decreases with decreasing temperature. This trend is opposite to that commonly observed in bulk silicon semiconductors, where reduced phonon scattering leads to higher mobility and increased current. Instead, it is consistent with the characteristics of ballistic-transport-induced avalanche.

This behavior arises because lower temperature suppresses thermal activation of interface trap states. As a result, fewer trapped carriers are released, which effectively reduces the active carrier concentration in the channel and weakens the current drive capability. In addition, in two-dimensional material systems, carrier mobility is often limited by interface scattering rather than bulk phonon scattering. When high-quality van der Waals interfaces are present, the positive effect of reduced temperature on mobility within this range can be relatively weak. In contrast, thermal activation effects, such as Fermi-level broadening and enhanced carrier injection efficiency, may dominate and lead to an apparent increase in current. Moreover, this temperature-dependent behavior indirectly reflects the stability of the device near room temperature. The output current shows only minor variation with temperature fluctuations. This stability is critical for practical circuit applications.

In avalanche photodetectors based on two-dimensional materials, thermal effects primarily influence device performance through their impact on carrier energy distribution and fluctuation dynamics, rather than through macroscopic temperature rise alone. Local carrier heating induced by high electric fields and impact ionization modifies the energy relaxation processes, which directly affects the statistical properties of carrier multiplication.

From a physical perspective, temperature-dependent variations in carrier mobility, scattering rates, and ionization probability manifest themselves as fluctuations in current and multiplication gain, which are most appropriately characterized in the frequency domain. As a result, thermal effects are intrinsically reflected in the noise power spectral density, where the combined influence of carrier heating, electro-thermal feedback, and stochastic impact ionization can be quantitatively assessed.

Therefore, the noise spectrum serves as a physically meaningful and application-relevant metric that naturally integrates thermal effects with transport and avalanche dynamics. This approach enables a unified evaluation of thermal management, carrier heating, and noise behavior within a single electro-thermal–noise framework, which is particularly suitable for ballistic avalanche operation in 2D heterostructures.

**Figure 9:**
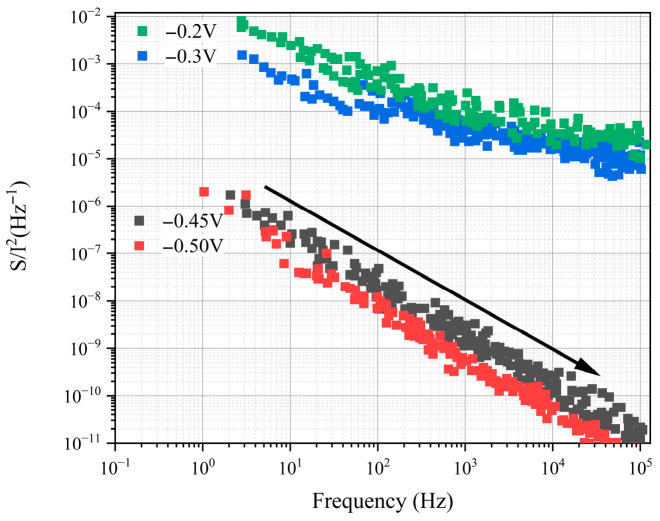
Change in normalized noise power spectral density with frequency for the vertically stacked metal-2D-material-metal structure.

Figure 9 shows the relationship between the normalized noise power spectral density and frequency for a vertically stacked metal-2D-metal structure under different bias voltages.

Based on the normalized noise power spectral density versus frequency relationship shown in the figure, a detailed analysis of the noise characteristics of the vertically stacked metal-2D-material-metal photodetector proposed in this paper can be conducted. As illustrated, in the double logarithmic coordinate system, the noise spectrum for all bias voltages exhibits a significant rise in the low-frequency range, followed by a monotonic decline as the frequency increases, eventually reaching a lower noise plateau in the high-frequency region. This behavior is a characteristic feature of the combination of low-frequency excess noise and white noise. Notably, at the two avalanche threshold biases of −0.45 V and −0.50 V, the noise spectrum shows a typical 1/f noise behavior over a wide frequency range, providing key insights into carrier dynamics under ballistic transport conditions.

This noise characteristic is highly consistent with the ballistic avalanche mechanism described in this paper. At lower bias voltages, the device operates in a linear or weakly nonlinear region, where carrier transport is predominantly quasi-ballistic with fewer scattering events, resulting in a generally low normalized noise level. As the bias voltage increases to the avalanche threshold, the internal electric field becomes strong enough to impart sufficient energy to the carriers within their mean free path, triggering deterministic collision ionization. This process is not the random avalanche multiplication chain typical of conventional APDs; instead, the statistical fluctuations in carrier multiplication are suppressed, which is reflected in the noise spectrum as 1/f noise. This indicates that the noise source contains both low-frequency components arising from trap-assisted tunneling or interface fluctuations and components from the suppressed avalanche randomness. The latter is partially “tamed” due to the deterministic nature of ballistic transport, resulting in a flatter noise spectrum decay compared to traditional APDs.

Further, the noise in the high-frequency region tends to a constant background level, which may be dominated by thermal noise or shot noise. Near the avalanche threshold, this background noise does not show the sharp increase seen in conventional APDs, where multiplication noise typically diverges due to avalanche gain fluctuations. This again confirms that the ballistic avalanche process has lower gain fluctuations. It should be noted that thermal management plays a critical role in this noise characteristic. Since carrier acceleration and collision ionization generate significant Joule heat, the accumulation of this heat may raise the local temperature of the device, thereby affecting the noise spectrum, especially under high-power operating conditions [18]. To mitigate the adverse impact of temperature rise on device performance, thermal management strategies are essential during design. We have considered the coupling of thermoelectric effects in our simulations and included noise components arising from thermal effects in the noise spectrum analysis. By optimizing the device’s thermal management, additional noise caused by localized overheating can be effectively reduced, further improving the low-frequency noise characteristics.

This noise spectrum strongly supports the core argument of this paper: axial ballistic transport and electric field confinement achieved through the vertically stacked structure can effectively reduce the excess noise factor in the avalanche multiplication process. Additionally, proper thermal management design helps suppress the noise enhancement effects caused by thermal accumulation, further improving the device’s noise performance and stability. This unique noise characteristic, combined with the previously discussed low operating voltage and high gain, gives this device architecture significant performance potential and competitiveness in low-light detection and high-frequency optoelectronic applications, compared to traditional vertical APDs. Considering manufacturing feasibility and reliability, interface state control and effective thermal management are key to reducing low-frequency noise and enhancing the long-term stability of the device. Future work should integrate in situ thermal simulation and noise modeling to optimize 2D material layer stacking and electrode design, driving the practical application of this architecture in high-sensitivity photodetection fields.

The vertically stacked metal-2D-metal structure proposed in the section introduces a new approach to avalanche photodetectors, leveraging two-dimensional materials such asBP and indium selenide. This configuration stands in contrast to the conventional vertical APD designs, which typically rely on bulk semiconductor materials. By stacking the 2D materials in a vertical arrangement, the device benefits from the unique electronic properties of the 2D materials, such as their high carrier mobility and minimal scattering in the ballistic transport regime. This leads to a more efficient avalanche process with lower threshold voltages and reduced noise compared to traditional APDs. Additionally, the vertical structure enhances the electric field distribution, allowing for more precise control of the avalanche process, which is crucial for achieving high gain and sensitivity at low power consumption.

When comparing this 2D-based vertical stacking technique to existing vertical APD technology, the former presents significant advantages in terms of device miniaturization, low-voltage operation, and noise reduction. Traditional vertical APDs often suffer from high bias requirements due to their reliance on bulk materials, which lead to larger device sizes and higher power consumption. These devices also exhibit significant noise due to the stochastic nature of impact ionization in bulk materials. In contrast, the 2D-based approach reduces the operating voltage by enabling ballistic transport in materials with nanoscale dimensions, where carriers experience minimal scattering and the avalanche process becomes more deterministic. This results in lower noise levels and higher performance, particularly in low-power applications such as quantum communication and single-photon detection.

In terms of manufacturability, the 2D material-based vertical structures face some challenges that are distinct from traditional APDs. The primary concern revolves around the integration of 2D materials with metal contacts, which is essential for creating high-quality interfaces. The contact quality and the potential for defects at the interface between the 2D materials and the metal contacts are critical for device performance [44]. If the contact resistance is too high, it can significantly degrade the device’s efficiency. Moreover, the fabrication of atomically thin 2D materials, while increasingly feasible, still presents challenges in terms of uniformity, scalability, and integration with existing semiconductor fabrication processes. However, advancements in van der Waals stacking and interlayer coupling techniques have made significant strides in overcoming these challenges, allowing for the reliable production of 2D material heterostructures. Furthermore, the potential for low-cost manufacturing methods, such as roll-to-roll processing or chemical vapor deposition, could make 2D-based vertical APDs more commercially viable in the future.

However, we believe that these issues can be effectively resolved through rational interface engineering design and material optimization in the future [25]. While the vertical stacking provides an inherent advantage in terms of heat dissipation due to the alignment of the heat flow with the electrodes, ensuring long-term stability under operating conditions remains a concern. The interface between the 2D materials and the metals must be carefully engineered to avoid degradation due to factors such as interlayer slip, defects, and thermal expansion mismatches. These factors could affect the long-term reliability and performance of the devices. Nonetheless, with proper interface engineering and material optimization, these issues can be mitigated.

On the basis of the above analysis, while the vertically stacked metal-2D-metal structure offers numerous advantages over traditional APDs in terms of performance, miniaturization, and power efficiency, it faces unique challenges in terms of manufacturing complexity and long-term reliability. However, the promising properties of 2D materials, coupled with ongoing advancements in fabrication techniques, suggest that this approach could represent the future of high-performance, low-power avalanche photodetectors.

## 4. Conclusions

In summary, this study systematically constructs a physical simulation model based on the Silvaco TCAD platform and achieves accurate simulation and mechanistic analysis of ballistic-transport-induced avalanche effects in two-dimensional InSe/BP heterojunctions. The accuracy of the model is first evaluated by comparison with key experimental data reported in the literature. The results demonstrate that the model can reliably reproduce core features such as low threshold voltage, high avalanche gain, and the unique positive temperature coefficient. These results indicate that the model effectively captures nearly scattering-free ballistic carrier transport at the nanoscale and the resulting deterministic impact ionization process. Through parameter scanning and mechanism analysis, the roles of gate electric field modulation and doping concentration optimization in controlling the avalanche threshold and multiplication gain are systematically clarified. The results reveal the physical essence of deterministic carrier acceleration and multiplication achieved through band engineering within sub-mean-free-path transport distances.

In addition, based on the validated physical model, this study innovatively proposes a vertically stacked metal–two-dimensional material–metal device architecture and predicts its enhanced avalanche performance. Simulation results show that this structure can further reduce the operating voltage, improve gain stability, and demonstrate the potential for room-temperature operation by optimizing the electric field distribution, enhancing carrier injection efficiency, and improving thermal management. This structural design provides a practical and feasible solution for overcoming the high operating voltage, high noise, and performance limitations of avalanche photodetectors based on conventional bulk materials.

Furthermore, this work provides a solid theoretical basis and a reliable simulation framework for the optimized design of high-performance and low-power avalanche optoelectronic devices. Future research can focus on precise control of interface engineering, exploration of different two-dimensional material combinations, and integration with practical fabrication processes, to advance these devices toward real-world applications [45,46,47].

## Figures and Tables

**Table 1:** The comparative table summarizing the key differences between conventional bulk APDs and ballistic 2D APDs [19].

Device/Structure/Material	R (A/W)	RT (ms)	λ (nm)	I_Dark_ (A)	EQE	NPD (W^−1^)	M	T (K)
BP/InSe APD	80	--	4000	--	248	--	10^4^–10^5^	10–180
BP APD	130	--	500–1100	2 × 10^−6^	310	6.5 × 10^7^	7	300
InSe APD	11,000	1	405–785	5 × 10^−9^	--	2.5 × 10^2^	500	--
MoTe_2_-WS_2_-MoTe_2_ APD	6.02	475	400–700	9.3 × 10^−11^	14.1	6.47 × 10^9^	587	295
Si	0.5–1.0	10^−6^	400–1100	10^−15^–10^−12^	50–80	--	50–100	300
Ge	0.7–1.2	10^−5^	800–1800	10^−15^–10^−12^	40–70	--	40–80	300
InGaAs	0.9–1.5	10^−6^	900–1700	10^−18^–10^−13^	70–90	--	30–50	300
HgCdTe	0.5–1.2	10^−6^	2000–18,000	10^−18^–10^−12^	30–70	--	100–200	77

## Data Availability

The original contributions presented in this study are included in the article. Further inquiries can be directed to the first author.
